# Six NSCL/P Loci Show Associations With Normal-Range Craniofacial Variation

**DOI:** 10.3389/fgene.2018.00502

**Published:** 2018-10-25

**Authors:** Karlijne Indencleef, Jasmien Roosenboom, Hanne Hoskens, Julie D. White, Mark D. Shriver, Stephen Richmond, Hilde Peeters, Eleanor Feingold, Mary L. Marazita, John R. Shaffer, Seth M. Weinberg, Greet Hens, Peter Claes

**Affiliations:** ^1^Department of Electrical Engineering, ESAT/PSI, KU Leuven, Leuven, Belgium; ^2^Medical Imaging Research Center, UZ Leuven, Leuven, Belgium; ^3^Department of Oral Biology, Center for Craniofacial and Dental Genetics, School of Dental Medicine, University of Pittsburgh, Pittsburgh, PA, United States; ^4^Department of Human Genetics, University Hospitals Leuven, Leuven, Belgium; ^5^Department of Anthropology, The Pennsylvania State University, University Park, PA, United States; ^6^Applied Clinical Research and Public Health, School of Dentistry, Cardiff University, College of Biomedical and Life Sciences, Heath Park, Cardiff, United Kingdom; ^7^Department of Human Genetics, Graduate School of Public Health, University of Pittsburgh, Pittsburgh, PA, United States; ^8^Department of Otorhinolaryngology, University Hospitals Leuven, Leuven, Belgium

**Keywords:** craniofacial, morphology, NSCL/P, candidate gene, ALSPAC

## Abstract

**Objectives:** Orofacial clefting is one of the most prevalent craniofacial malformations. Previous research has demonstrated that unaffected relatives of patients with non-syndromic cleft lip with/without cleft palate (NSCL/P) show distinctive facial features, which can be an expression of underlying NSCL/P susceptibility genes. These results support the hypothesis that genes involved in the occurrence of a cleft also play a role in normal craniofacial development. In this study, we investigated the influence of genetic variants associated with NSCL/P on normal-range variation in facial shape.

**Methods:** A literature review of genome wide association studies (GWAS) investigating the genetic etiology of NSCL/P was performed, resulting in a list of 75 single nucleotide polymorphisms (SNPs) located in 38 genetic loci. Genotype data were available for 65 of these selected SNPs in three datasets with a combined sample size of 7,418 participants of European ancestry, whose 3D facial images were also available. The effect of each SNP was tested using a multivariate canonical correlation analysis (CCA) against 63 hierarchically-constructed facial segments in each of the three datasets and meta-analyzed. This allowed for the investigation of associations between SNPs known to be involved in NSCL/P and normal-range facial shape variations in a global-to-local perspective, without preselecting specific facial shape features or characteristics.

**Results:** Six NSCL/P SNPs showed significant associations with variation in normal-range facial morphology. rs6740960 showed significant effects in the chin area (*p* = 3.71 × 10^−28^). This SNP lies in a non-coding area. Another SNP, rs227731 near the *NOG* gene, showed a significant effect in the philtrum area (*p* = 1.96 × 10^−16^). Three SNPs showed significant effects on the shape of the nose. rs742071 (*p* = 8.71 × 10^−14^), rs34246903 (*p* = 6.87 × 10^−12^), and rs10512248 (*p* = 8.4 × 10^−9^). Respectively, these SNPs are annotated to *PAX7, MSX1*, and *PTCH1*. Finally, rs7590268, an intron variant of *THADA*, showed an effect in the shape of the supraorbital ridge (*p* = 3.84 × 10^−7^).

**Conclusions:** This study provides additional evidence NSCL/P-associated genetic variants influence normal-range craniofacial morphology, with significant effects observed for the chin, the nose, the supraorbital ridges and the philtrum area.

## Introduction

Human facial features are highly variable and mostly genetically determined. Although craniofacial morphology is of interest to many scientists, its genetic architecture remains poorly understood (Roosenboom et al., 2016). Clinical dysmorphologists have hypothesized that genes responsible for syndromes with a distinctive facial phenotype are also involved in normal craniofacial development (Winter, 1996). Additionally, many loci found in genome wide association studies on human facial morphology were involved in syndromes affecting the face (Adhikari et al., 2016; Shaffer et al., 2016; Claes et al., 2018). Hence, investigating genes involved in craniofacial disorders constitutes a suitable approach to help unravel the genetic architecture of facial morphology.

With an incidence of 1.7 in 1,000 livebirths, non-syndromic cleft lip with or without cleft palate (NSCL/P) is one of the most common craniofacial anomalies, causing significant functional and psychological burden to the patient (Mossey et al., 2009). NSCL/P has a multifactorial etiology, which includes both genetic and environmental factors. Although the genetic background of NSCL/P is complex, significant progress has been made in the identification of NSCL/P susceptibility loci through genetic association studies (references listed in Table 1).

**Table 1:** Overview of lead-SNPs from the literature survey.

**Region**	**Lead SNP**	**Location (bp)**	***p*-value**	**Population**	**Method**	**References**
1p22	rs560426	94553438	5.01E-12	Asian + European	GWAS	Beaty et al., 2010
			3.14E-12	Asian + European	Meta-analysis	Ludwig et al., 2012
	rs481931	94570016	1.06E-12	Chinese	Meta-analysis	Yu et al., 2017
	rs4147803	94582293	7.97E-12	Chinese	Meta-analysis	Yu et al., 2017
	rs66515264	94558110	4.14E-17	Multi-ethnic	Meta-analysis	Leslie et al., 2017
1p36	rs742071	18979874	7.02E-09	Asian + European	Meta-analysis	Ludwig et al., 2012
	rs4920524	18978372	3.72E-09	Multi-ethnic	Meta-analysis	Leslie et al., 2016
	rs9439713	18972776	6.02E-13	Multi-ethnic	Meta-analysis	Leslie et al., 2017
1q32	rs861020	209977111	3.24E-12	Asian + European	Meta-analysis	Ludwig et al., 2012
			1.3E-14	Chinese	Meta-analysis	Yu et al., 2017
	rs2235371	209964080	8.69E-22	Chinese	Meta-analysis	Sun et al., 2015
	rs1044516	209959614	6.57E-13	Chinese	Meta-analysis	Sun et al., 2015
	rs596731	209993801	3.77E-10	Chinese	Meta-analysis	Sun et al., 2015
	rs742214	209960925	1.62E-19	Chinese	Meta-analysis	Sun et al., 2015
	rs2064163	210048819	8.6E-19	Chinese	Meta-analysis	Yu et al., 2017
	rs642961	209989270	2.76E-15	Chinese	Meta-analysis	Yu et al., 2017
	rs9430019	210050794	1.68E-12	Chinese	Meta-analysis	Yu et al., 2017
2p21(*THADA*)	rs7590268	43540125	1.25E-08	Asian + European	Meta-analysis	Ludwig et al., 2012
2p21(*PKDCC*)	rs6740960	42181679	5.71E-13	Multi-ethnic	Meta-analysis	Ludwig et al., 2017
2p24.2	rs7552	16733928	4.22E-08	Multi-ethnic	Meta-analysis	Leslie et al., 2016
			5.83E-22	Chinese	Meta-analysis	Yu et al., 2017
	rs7566780	16729357	4.28E-09	Multi-ethnic	Meta-analysis	Leslie et al., 2017
	rs10172734	16733054	2.89E-20	Chinese	Meta-analysis	Yu et al., 2017
2p25.1	rs287980	9971366	1.94E-08	Chinese	Meta-analysis	Yu et al., 2017
3p11.1	rs7632427	89534377	3.9E-08	Asian + European	Meta-analysis	Ludwig et al., 2012
3q28	rs76479869	189553372	1.16E-08	Multi-ethnic	Meta-analysis	Leslie et al., 2017
3q29	rs338217	2979676	9.70E-10	European	Mega-analysis	Mostowska et al., 2018
4p16.2	rs34246903	4794195	4.45E-08	Chinese	Meta-analysis	Yu et al., 2017
	rs1907989	4818925	1.58E-08	Chinese	Meta-analysis	Yu et al., 2017
4q28.1	rs908822	124906257	4.33E-08	Chinese	Meta-analysis	Yu et al., 2017
5p12	rs10462065	44068846	1.12E-08	Chinese	Meta-analysis	Yu et al., 2017
6p24.3	rs9381107	9469238	2.72E-09	Chinese	Meta-analysis	Yu et al., 2017
8p11.23	rs13317	38269514	3.96E-08	Chinese	Meta-analysis	Yu et al., 2017
8q21	rs12543318	88868340	1.9E-08	Asian + European	Meta-analysis	Ludwig et al., 2012
			8.8E-12	Chinese	Meta-analysis	Yu et al., 2017
			8.75E-12	Multi-ethnic	Meta-analysis	Leslie et al., 2017
	rs1034832	88918331	1.35E-10	Chinese	Meta-analysis	Yu et al., 2017
8q22.1	rs957448	95541302	9.6E-13	Chinese	Meta-analysis	Yu et al., 2017
	rs12681366	95401265	2.35E-10	Chinese	Meta-analysis	Yu et al., 2017
8q24	rs987525	129946154	1.11E-16	Asian + European	GWAS	Beaty et al., 2010
			3.41E-10	Central European	GWAS	Birnbaum et al., 2009
			9.18E-10	European	GWAS	Grant et al., 2009
			Not reported	European	GWAS	Mangold et al., 2010
			5.12E-35	Asian + European	Meta-analysis	Ludwig et al., 2012
	rs7845615	129888794	1.03E-10	Chinese	Meta-analysis	Yu et al., 2017
	rs7017252	129950844	8.47E-16	Chinese	Meta-analysis	Yu et al., 2017
	rs55658222	129976136	8.3E-44	Multi-ethnic	Meta-analysis	Leslie et al., 2017
9q22.2	rs7871395	92209587	6.06E-09	Chinese	Meta-analysis	Yu et al., 2017
9q22.32	rs10512248	98259703	5.1E-10	Chinese	Meta-analysis	Yu et al., 2017
10q25	rs7078160	118827560	1.07E-07	Asian + European	GWAS	Beaty et al., 2010
			1.92E-08	European	GWAS	Mangold et al., 2010
			3.96E-11	Asian + European	Meta-analysis	Ludwig et al., 2012
			3.09E-10	Chinese	Meta-analysis	Sun et al., 2015
	rs6585429	118893231	7.14E-13	Chinese	Meta-analysis	Yu et al., 2017
12q13.13	rs3741442	53346750	3.72E-12	Chinese	Meta-analysis	Yu et al., 2017
12q13.2	rs705704	56435412	1.29E-09	Chinese	Meta-analysis	Yu et al., 2017
12q21.1	rs2304269	72080272	1.32E-12	Chinese	Meta-analysis	Yu et al., 2017
	rs7967428	72089040	3.08E-12	Chinese	Meta-analysis	Yu et al., 2017
13q31.1	rs9545308	80639405	2E-09	Chinese	Meta-analysis	Yu et al., 2017
	rs8001641	80692811	2.62E-10	Asian + European	Meta-analysis	Ludwig et al., 2012
	rs11841646	80679302	3.62E-10	Multi-ethnic	Meta-analysis	Leslie et al., 2017
14q22.1	rs7148069	51839645	1.69E-08	Chinese	Meta-analysis	Yu et al., 2017
	rs4901118	51856109	6.94E-10	Multi-ethnic	Meta-analysis	Ludwig et al., 2017
14q32.13	rs1243573	95379583	8.61E-10	Chinese	Meta-analysis	Yu et al., 2017
15q13	rs1258763	33050423	8.13E-14	European	Meta-Analysis	Ludwig et al., 2016
15q22.2	rs1873147	63312632	2.81E-08	European	Meta-analysis	Ludwig et al., 2012
15q24	rs28689146	75005575	6.61E-09	Multi-ethnic	Meta-analysis	Ludwig et al., 2017
	rs11072494	74889163	2.4E-08	Multi-ethnic	Meta-analysis	Leslie et al., 2017
16p13.3	rs8049367	3980445	8.98E-12	Chinese	Meta-analysis	Sun et al., 2015
	rs2283487	3969886	1.27E-10	Chinese	Meta-analysis	Yu et al., 2017
	rs17136624	3996282	3.82E-10	Chinese	Meta-analysis	Yu et al., 2017
17p13.1	rs9788972	8919630	7.05E-09	Asian + European	GWAS	Beaty et al., 2010
	rs4791774	8930220: 8930232	5.05E-19	Chinese	Meta-analysis	Sun et al., 2015
	rs11273201	8930225	7.84E-12	Multi-ethnic	Meta-analysis	Leslie et al., 2016
	rs7406226	8914693	1.46E-08	Central/ South American	Meta-analysis	Leslie et al., 2016
	rs2872615	8929845	8.81E-12	Chinese	GWAS	Yu et al., 2017
	rs1880646	8929845	1.69E-11	Chinese	GWAS	Yu et al., 2017
	rs12944377	8947708	8.23E-21	Multi-ethnic	Meta-analysis	Leslie et al., 2017
17q21.32	rs4968247	44988703	8.7E-10	Chinese	GWAS	Yu et al., 2017
	rs1838105	45008935	1.31E-11	Chinese	GWAS	Yu et al., 2017
17q22	rs227731	54773238	1.07E-08	European	GWAS	Mangold et al., 2010
			1.87E-09	Asian + European	Meta-analysis	Ludwig et al., 2012
			8.83E-09	Chinese	Meta-analysis	Yu et al., 2017
			1.77E-09	Multi-ethnic	Meta-analysis	Leslie et al., 2017
17q23.2	rs1588366	61076428	1.41E-08	European	Meta-analysis	Leslie et al., 2016
19p13.3	rs3746101	2050823	2.44E-08	European	Meta-analysis	Ludwig et al., 2017
19q12	rs73039428	33521150	2.92E-08	Multi-ethnic	Meta-analysis	Leslie et al., 2016
20q12	rs13041247	39269074	1.44E-11	Asian + European	GWAS	Beaty et al., 2010
			6.17E-09	Asian + European	Meta-analysis	Ludwig et al., 2012
	rs6129653	39275603	8.57E-12	Chinese	Meta-analysis	Yu et al., 2017
	rs6072081	39261054	1.87E-12	Multi-ethnic	Meta-analysis	Leslie et al., 2017

It has previously been hypothesized that NSCL/P genes can have an effect on normal facial morphology. Boehringer et al. (2011) found associations between genetic loci involved in NSCL/P and normal craniofacial traits, namely bizygomatic distance and nose width. Furthermore, several studies have found that unaffected relatives of patients with NSCL/P show distinctive facial characteristics in comparison to a control group, such as midfacial retrusion and broadening of the upper face, which could be defined as endophenotypes (Weinberg et al., 2008; Roosenboom et al., 2015).

In this study, we investigated the involvement of NSCL/P-associated genetic variants in normal-range facial variation. We used a candidate variant approach in combination with a new approach to study facial phenotypes based on spatially-dense, data-driven, global-to-local segmentations of facial 3D images. This segmentation approach was adopted from Claes et al. (2018) and offered two advantages. First, it allowed us to study the effects of candidate variants on facial shape in a hierarchical manner, providing complete coverage of the 3D facial surface at different levels of scale. Second, it allowed for an open-ended data-driven approach to establishing phenotypes, thereby avoiding preselection of phenotypic measurements. The latter aspect is in contrast to other cleft candidate gene studies (Boehringer et al., 2011; Liu et al., 2012) in which preselected phenotypic traits on a normal-range sample were studied.

## Materials and methods

### Sample and recruitment details

#### Pittsburgh dataset

Three datasets with 3D images and corresponding genetic data were used in this study. For the Pittsburgh sample, data were obtained from the 3D Facial Norms Database, which is a repository of 3D facial images and measurements (Weinberg et al., 2016). Participants were recruited in Pittsburgh, PA; Seattle, WA; Houston, TX; and Iowa City, IA. This dataset consists of 2,382 3D images with corresponding covariates of sex, age, weight, height, and genotype data. Participants ranged from 3 to 40 years old (mean age = 22; SD age = 9) and were of self-reported European ancestry. Individuals were excluded if they reported a personal or family history of any birth defect or syndrome affecting the head or face, a personal history of any significant trauma, surgery, or any medical condition that might alter the structure of the face. Of 2,382 participants with 3D images and covariate data, 42 were excluded based on having poor 3D image quality and 22 were excluded because of missing data on covariates. Based on the genotype data, relatives (*n* = 10) and genetic PCA outliers (*n* = 15) were identified and removed. The intersection of individuals with quality-controlled images, covariates and genotype data included 2,297 subjects.

#### Penn state dataset

The data collected through Penn State consists of participants recruited in State College, PA; New York, NY; Urbana-Champaign, IL; Twinsburg, OH; Dublin, Ireland; Rome, Italy; Warsaw, Poland; and Porto, Portugal. The minimum age in this sample was 18, the maximum age 83 (mean age = 29; SD age = 14). Data on self-reported ancestry, body characteristics, age and sex as well as genotype data were obtained. Individuals were excluded if they reported a personal or family history of any birth defect or syndrome affecting the head or face, a personal history of any significant trauma, surgery, or any medical condition that might alter the structure of the face. From the entire Penn State dataset (*n* = 6,588), participants were excluded based on missing covariate data (*n* = 748) and quality control of the images resulted in the elimination of 52 participants. Based on the genotype data, European participants were selected (*n* = 1,614) and related individuals were removed (*n* = 59) (see Genotyping methods section), resulting in 1,555 participants for analysis. No genetic outliers were identified.

#### ALSPAC dataset

The Avon Longitudinal Study of Parents and their Children (ALSPAC) is a longitudinal birth cohort in which pregnant women residing in Avon with an expected delivery date between 1 April 1991 and 31 December 1992 were recruited (Boyd et al., 2013; Fraser et al., 2013). At the time 14,541 pregnant women were recruited and DNA samples were collected for 11,343 children. Please note that the study website contains details of all the data that is available through a fully searchable data dictionary.^1^ Genome-wide data was available for 8,952 subjects of the B2261 study which is titled “Exploring distinctive facial features and their association with known candidate variants.” In addition to this, 4,731 3D-images were available along with corresponding data files containing information about sex, age, weight, height, ancestry, and other body characteristics. Participant ages ranged from 14 to 17 years old (mean age = 15; SD age = 0.5). Image quality control analysis resulted in the removal of 14 images of poor quality. 199 participants were removed due to self-reported non-European ancestry, 168 participants were removed because of missing covariate data and 726 individuals were removed because of relatedness. No genetic outliers were identified. The intersection of participants with quality-controlled images, covariates and genotype data included 3,566 individuals.

### Genotyping

#### Genotype quality control and population structure

For both the Pittsburgh and Penn State sample, the genotype data were obtained as described in Claes et al. (2018). Pittsburgh participants were genotyped at the Center for Inherited Disease Research (CIDR) at Johns Hopkins University on the Illumina OmniExpress+ Exome v1.2 array plus 4,322 investigator-chosen SNPs included to capture variation in specific regions of interest based on previous studies of the genetics of facial variation (Shaffer et al., 2016). Genotypes were imputed to the 1000 Genomes Project Phase 3 reference panel (The 1000 Genomes Project Consortium et al., 2015), using SHAPEIT2 (Delaneau et al., 2013) for prephasing of haplotypes and IMPTUE2 for the imputation (Howie et al., 2009, 2011). Participants in the Penn State sample were either genotyped on the Illumina Human Hp200c1 BeadChip (IRB 32341) or the 23andMe v3 and v4 arrays (IRB 44929, 13103, 2503, 4320). In individuals with more than 500,000 variants, genotypes were prephased with SHAPEIT2 (Delaneau et al., 2013) and imputed to the 1000 Genomes Phase 3 reference using the Sanger Imputation Server^2^ with the Positional Burrows-Wheeler Transform (PBWT) pipeline (Durbin, 2014). For the ALSPAC sample, the participants were genotyped using the Illumina HumanHap550 quad genome-wide SNP genotyping platform by Sample Logistics and Genotyping Facilities at the Wellcome Trust Sanger Institute (Cambridge, UK) and the Laboratory Corporation of America (Burlington, NC, US), supported by 23andMe. Haplotypes were estimated using SHAPEIT2 (Delaneau et al., 2013) and imputed to the 1000 genomes reference panel (Phase 1, Version 3; Abecasis et al., 2012) using IMPUTE2 (Howie et al., 2009, 2011).

To select participants with primarily European ancestry in the Penn State Sample, an ADMIXTURE analysis was done with the 1000 Genomes Phase 3 dataset as the reference (Alexander et al., 2009). The estimated number of populations (*k*) was 5, determined by the cross-validation (CV) error for each *k* value. These results were then used to select samples with < 10% ancestry from all of the non-European admixture components. In the ASPAC sample, population stratification was assessed by multidimensional scaling analysis and compared with Hapmap II (release 22) European descent (CEU), Han Chinese, Japanese and Yoruba reference populations; all individuals with non-European ancestry were removed.

To assess population structure within the European subsets after removing non-European individuals, the same protocol was followed for the three datasets using PLINK 1.9 (Purcell et al., 2007). First, SNPs with a minor allele frequency (MAF) < 5% or more than 5% genotype data missing, were filtered out. Subsequently, SNPs were pruned for linkage disequilibrium (LD) with *r*^2^ set at 0.2 in a pairwise manner, with a moving window size of 50 variants shifting 5 variants each step. Subsequently, related individuals were identified and removed when the proportion of identity by descent (IBD) was higher than 0.125. Ancestry axes were determined with principal component analysis (PCA). Outliers (*n* = 15) were removed based on Z-scores calculated in the first 10 principal components. Z-scores higher than 6 indicated outliers, who were subsequently removed after which PCA was computed again.

#### Candidate variant selection

To select a set of NSCL/P candidate variants, 12 GWAS studies investigating the genetic etiology of NSCL/P were reviewed. Table 1 is a list of NSCL/P associated SNPs that have been selected based on a genome-wide significant association in at least one of the 12 GWAS studies. LD data for SNPs of the same locus were collected using the NCI NIH analysis tool LDmatrix in LDlink in the European populations (CEU, TSI, FIN, GBR, and IBS; Machiela and Chanock, 2015). With this data, SNP-pairs in perfect LD (*r*^2^ = 1 and *D*′ = 1) were detected and one SNP per pair was eliminated to avoid unnecessary multiple testing. The elimination of SNPs in LD resulted in a set of 75 lead-SNPs from 38 different loci (Table 1). Genotypes of 10 of these SNPs were absent in either of the three datasets (highlighted in gray). Therefore, 65 SNPs from 34 loci had been included in the analysis.

### Phenotyping

#### Acquisition

Facial 3D surface images were acquired using two stereophotogrammetry systems and one laser scanning system. Facial surface data of the Pittsburgh sample were collected using the 3dMDface camera systems (3dMD, Atlanta, GA). For the Penn State sample, both 3dMDface camera systems and Vectra H1 (Canfield Scientific, Parsippany, NJ) camera systems were used. For the ALSPAC sample, a Konica Minolta Vivid 900 laser scanner (Konica Minolta Sensing Europe, Milton Keynes, UK) was used to take two high-resolution facial images which were subsequently processed, merged and registered using an algorithm implemented as a macro in Rapidform® software; INUS Technology Inc., Seoul, South Korea (Kau et al., 2004; Zhurov et al., 2005; Toma et al., 2008). Participants in all datasets were asked to have their mouth closed and to maintain a neutral facial expression during image capture.

#### Registration and quality control

3D surface images were imported into Matlab 2016b in.obj format to perform spatially dense registration (MeshMonk^3^). After importing the images, five positioning landmarks were indicated in the corners of the eye, the tip of the nose and the corners of the mouth to establish a crude alignment of the images. Subsequently, the images were cleaned by removing hair, ears, and any dissociated polygons. A symmetrical anthropometric mask (Claes et al., 2012) of 7,160 landmarks was then mapped onto the preprocessed images (Snyders et al., 2014). This resulted in homologous spatially dense configurations of quasi-landmarks.

After the registration, image quality control was performed to identify poorly remapped faces using two approaches. First, as described in Claes et al. (2018), outlier faces were identified by calculating Z-scores from the Mahalanobis distance between the mean face and each individual face. Faces with Z-scores higher than 2 were manually checked. Second, a score was calculated that reflects the missing data present in the image due to holes, spikes and other mesh artifacts that can be caused by facial hair or errors during the preprocessing steps, for example. Images with scores indicating a high amount of missing data, indicating large gaps in the mesh, were also manually checked. During the manual check, the images were either classified as images of poor quality or were preprocessed and mapped again.

The anthropometric mask (AM) is symmetrical relative to the sagittal plane, which allows reflected images to be created by changing the sign of the x-coordinate of the original mapped images. Both the original and the reflected remapped faces were then superimposed following a generalized Procrustes superimposition to eliminate differences in orientation, position and scale (Rohlf and Slice, 1990). Symmetrized images were created by averaging the original and the reflected images. All subsequent analyses were performed using these symmetrized images. Facial sizes were calculated by taking the centroid size of the spatially dense configurations.

#### Segmentation

**Figure 1:**
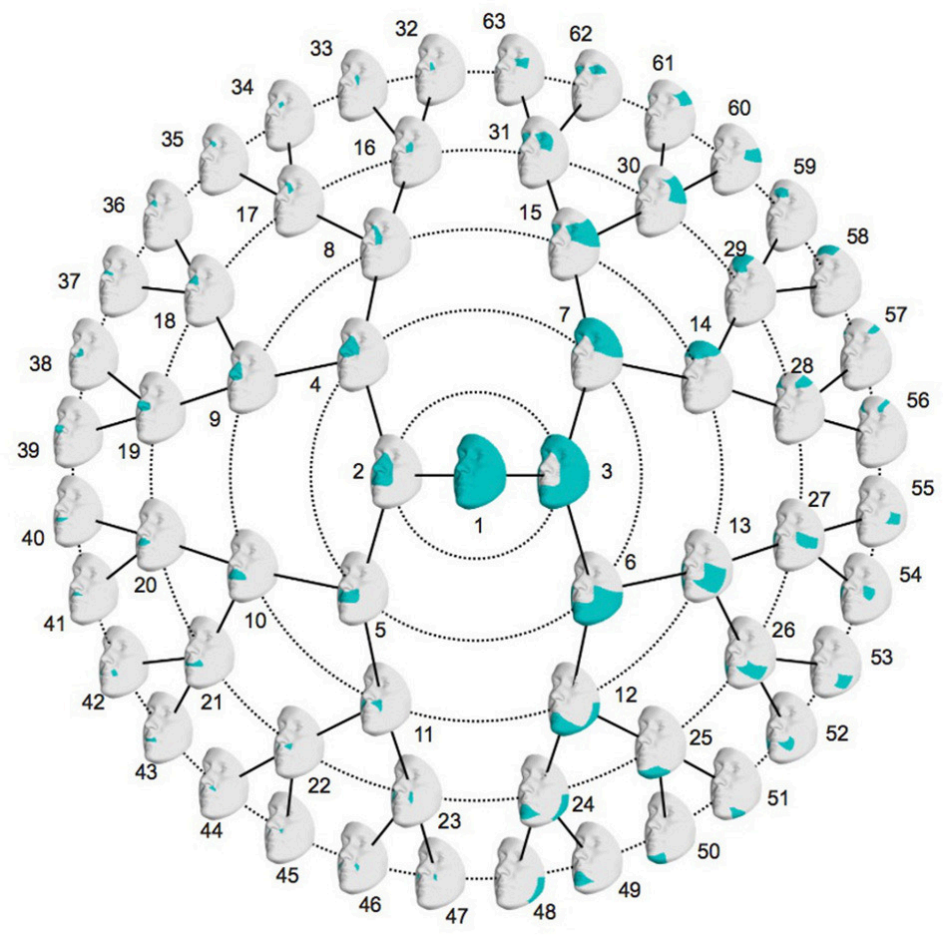
Global-to-local facial segmentation: 63 facial segments represented in blue. Starting from the global facial configuration in the center, the segment is split into two segments in level one, which are again split in two segments, which leads to four segments in level two. This is repeated up until level five which then contains 32 facial segments.

To study the effects of NSCL/P candidate variants on facial shape in a detailed manner, a data-driven facial segmentation was performed (Claes et al., 2018). First, the images were corrected for the confounding factors age, age-squared, sex, weight, height, facial size and the first four ancestry axes using partial least-squares regression (PLSR; function plsregress from Matlab 2016b). Because of potential systematic differences in genotyping platforms or imputation efforts, the genomic ancestry axes were calculated on each dataset separately. Following this trend, the images were corrected in each dataset separately. After the correction, the segmentation was performed on all three datasets combined. Facial segments were defined by grouping vertices that are strongly correlated and connected using hierarchical spectral clustering, as described in Claes et al. (2018). The strength of covariation between quasi-landmarks was defined using Escoufier's RV coefficient (Escoufier, 1973), which is a scalar measure of strength of association between two groups of variables and is used in morphometric studies on biological shapes (Klingenberg, 2009). The RV coefficient allowed us to build a structural similarity matrix that defined the hierarchical construction of 63 facial segments, consisting of five levels (Figure 1). Subsequently, all segments independently were aligned using generalized Procrustes superimposition. To capture the major variance in the facial segments with fewer variables, a PCA was performed on each of the 63 segments in combination with parallel analysis (Hayton et al., 2004). Parallel analysis can be used to eliminate noisy or meaningless shape variations that result from sources of error, such as 3D image acquisition and/or quasi-landmark registration, as described in Claes et al. (2018). After combining the three datasets a large sample size (*N* = 7,418) is obtained which is beneficial to obtain a well-defined segmentation of landmark covariations and the determination of significant principal components (PCs) using parallel analysis. Moreover, facial shape data across all three datasets are now in the same shape space, enabling across-dataset analyses including a meta-analysis.

### Meta-analysis

#### Statistical analysis

Each candidate SNP was tested against 63 facial segments each represented by multiple dimensions of variation (principal components). To this end we used canonical correlation analysis (CCA, canoncorr in Matlab 2016b) to test the effect of the 64 SNPs on facial shape under the reduced model. CCA is a multivariate testing framework which extracts the linear combination of PCs from a facial segment that has maximal correlation with the SNP being tested. The correlation is tested for significance based on Rao's F-test approximation (right tail, one-sided test; Olson, 1976). Using CCA, we tested each SNP individually under the additive genetic model in all three datasets. The reduced model was obtained after removing the linear effects of confounding factors age, age^2^, sex, weight, height, facial size and the first four ancestry axes using PLSR. Both the independent (SNP genotype) and the dependent (facial shape) variables were corrected for these covariates. Additionally, a partial least squares regression was performed (PLSR, plsregress in Matlab 2016b) in the same way as the CCA, to obtain the percentage of variance explained by the SNP in the facial segment that was tested.

#### Meta-analysis

To maximize the statistical power from the combination of the three datasets we performed a meta-analysis in a round-robin fashion. Using CCA, each of the three datasets was in turn used to discover and define the linear combination of phenotypic variables that is maximally correlated with the SNP. This step of the meta-analysis will be referred to as the discovery. Subsequently, the other two datasets were projected onto the loadings obtained from CCA (the linear combination of phenotypic variables), creating univariate phenotypic variables which are then also tested for genotype-phenotype associations. This step of the meta-analysis will be referred to as the replication. The replication results in univariate phenotypic variables are measured in the replication datasets as a function of the discovered phenotypic trait in the CCA. In doing so, as proposed in Claes et al. (2018), the phenotypic trait discovered in CCA can be explicitly measured in the replication datasets enabling the combination of the statistical results across all datasets. The univariate phenotypic scores were statistically tested for association in a standard linear regression with the SNP genotypes as independent variables (function regstats in Matlab2016b). This function employs a t-statistic and a positive-sided *p*-value was obtained with the Student's T cumulative distribution function (function tcdf in Matlab2016b; Devroye, 1986).

**Figure 2:**
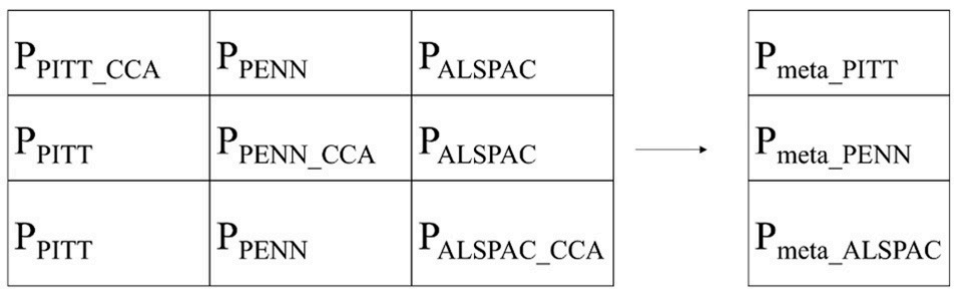
Schematic representation of *p*-values used in the meta-analysis.

After repeating the discovery plus two replications for each dataset in turn, we ended up with nine *p*-values (schematically represented in Figure 2). Each row contains a discovery *p*-value, determined by a multivariate CCA, and two replication *p*-values, each determined by a univariate linear regression. Row-wise each *p*-value was obtained from non-overlapping datasets and therefore independent and can be combined in a meta-analysis according to Stouffer et al.'s method 1949. This resulted in three meta-analyses *p*-values per segment, per SNP. Column-wise the *p*-values are not independent and therefore cannot be combined in the same manner.

#### Multiple testing correction

Analyzing 65 SNPs in 63 separate facial segments introduces a multiple testing burden. The facial segments are overlapping and hierarchically constructed and are thus not completely independent. To determine the number of independent tests, we evaluated the eigenvalues of pairwise multivariate correlations between facial segments and of pairwise genotype correlations and determined 37 independent segments (Li and Ji, 2005). Some of the SNPs tested are in high LD, which resulted in 56 independent genetic variants. The round-robin meta-analysis resulted in three *p*-values, for which we also corrected. Therefore, a total Bonferroni correction for the effective numbers of independent segments, SNPs and round-robin sequences resulted in an adjusted significance threshold of 8.04 × 10^−6^ [i.e., 0.05/(37 × 56 × 3)].

## Results

### Meta-analysis

The data-driven facial segmentation resulted in 63 facial segments that are hierarchically represented in Figure 1. At level two, four segments are identified, covering the nose, the mouth, the lower and the upper facial area, which coincides with the facial segmentation in Claes et al. (2018).

**Figure 3:**
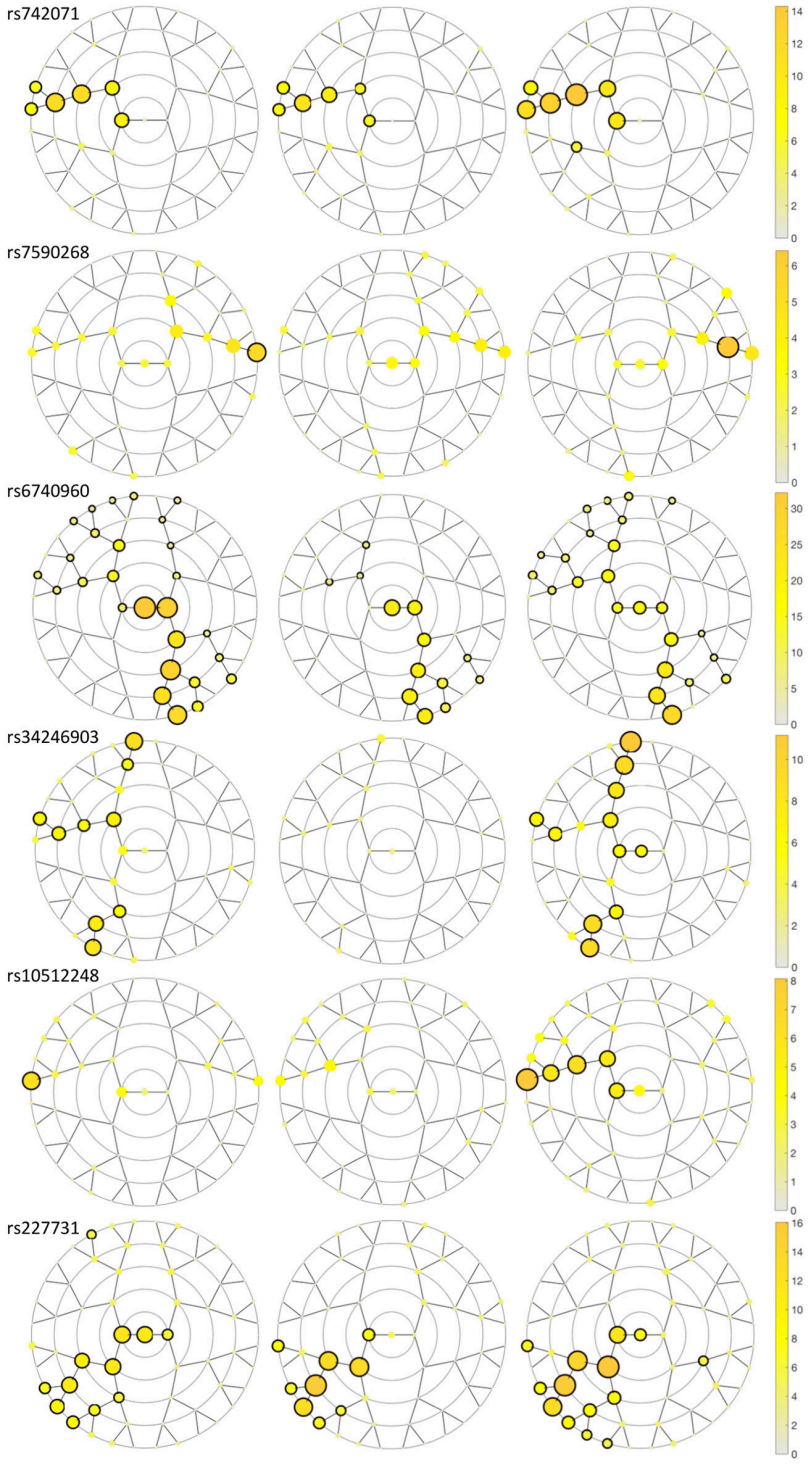
Meta-analyses results plotting the –log_10_ *p*-value for the SNPs in each segment in each discovery dataset meta-analysis. A black circle surrounding the yellow circle shows significance (*p*-value < 8.04 × 10^−6^) in the segment encircled in the meta-analysis. From left to right, the discovery datasets are Pittsburgh, Penn State and ALSPAC.

We identified six genetic loci involved in the etiology of NSCL/P that had significant effects on craniofacial morphology, namely rs742071 in 1p36, rs6740960, and rs7590268 in 2p21, rs34246903 in 4p16.2, rs10512248 in 9q22.32 and rs227731 in 17q22 (Table 2). These SNPs reached a *p*-value below the Bonferroni threshold of 8.04 × 10^−6^ in at least one facial segment in one of the three meta-analyses (Figure 3; Supplementary Figure 1). The percentage of variation explained by each SNP in a specific facial segment is reported in Table 2. Overall these percentages are low with the highest percentage of variation explained being 0.31% by rs6740960 in the chin area. Some variants, such as rs742071, rs6740960 and rs227731 show strong significance of association in the global segments as well as in the most local segments, with affected segments coming back in all three meta-analyses (Figure 3). Variants rs34246903 and rs10512248 both only show significant effects in the PITT and ALSPAC meta-analyses. Finally, rs7590268 is a variant that only shows effects in the local segments. Although there are no significant effects in the PENN STATE meta-analysis, the eyebrow region shows suggestive *p*-value signals.

**Table 2:** Discovery and meta-analysis results of significant SNPs in the most relevant segment.

**SNP**	**Region**	**Location (bp)**	**Candidate Gene**	**Alleles**	**MAF (1000G)**	**Segment**	**% Var Explained**	**CCA**		**Meta-Analysis**
								**CC**	***p*-value**	***p*-value**
rs742071	1p36	18979874	*PAX7*	T < G	0.432	19	0.077	0.1309	1.24E-02	1.73E-12
							0.083	0.1363	1.47E-01	2.82E-11
							0.110	0.1484	2.25E-08	8.71E-14
rs7590268	2p21	43540125	*THADA*	G < T	0.25	28	0.091	0.1382	3.49E-03	2.41E-05
							0.108	0.1470	5.28E-02	7.80E-05
							0.051	0.1042	1.48E-02	3.84E-07
rs6740960	2p21	42181679	*PKDCC*	A < T	0.483	49	0.310	0.2106	4.03E-13	5.49E-28
							0.226	0.1818	3.78E-04	1.27E-21
							0.142	0.1507	8.08E-09	3.71E-28
rs34246903	4p16.2	4794195	*MSX1*	C < A	0.325	32	0.115	0.0957	8.54E-03	1.43E-09
							0.070	0.0721	5.54E-01	5.00E-05
							0.248	0.1398	8.74E-12	6.87E-12
rs10512248	9q22.32	98259703	*PTCH1*	G < T	0.32	39	0.088	0.1135	1.31E-02	5.65E-07
							0.081	0.1125	4.14E-01	1.15E-04
							0.133	0.1317	4.64E-07	8.40E-09
rs227731	17q22	54773238	*NOG*	G < T	0.452	21	0.092	0.1188	5.51E-03	1.18E-11
							0.173	0.1633	2.45E-04	2.98E-16
							0.128	0.1334	5.33E-08	1.96E-16

*Minor allele < major allele. The risk allele is underlined. CC, canonical correlation. The CCA-correlations are listed in the order of Pittsburgh, Penn State and ALSPAC, the same order is given for the discovery p-values and the meta-analysis p-values*.

**Figure 4:**
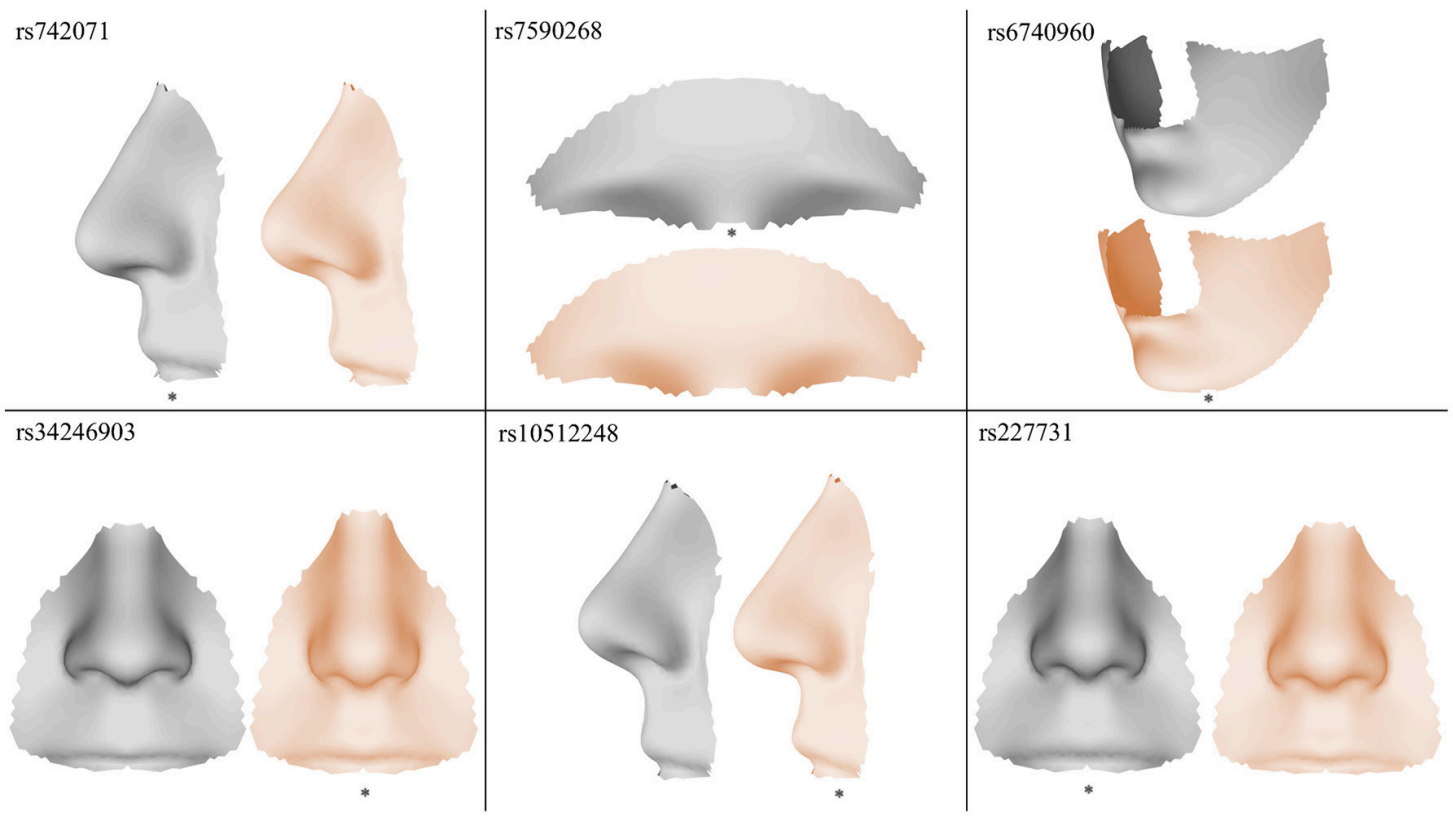
Visualization of the effects of the SNPs exaggerated in the direction of the minor allele (gray) and the major allele (orange), the risk allele is indicated with a *.

The results of the meta-analyses of the remaining SNPs are provided in Supplementary Table 1. rs742071 in 1p36 is associated with an effect on the shape of the tip of the nose, with the angle between the nose and the lip being decreased in association with the risk allele (Figure 4). Two other SNPs (rs4920524 and rs9439713) showed very similar effects (Supplementary Table 1) and were in high LD with rs742071 (Supplementary Table 2). rs7590268 in 2p21, showed a significant effect in the supraorbital ridge, with a more protruded and pronounced shape associated with the minor allele. Another SNP in 2p21, rs6740960, showed an effect on the shape of the chin, where the major allele is associated with a more protruded but shorter in length chin. rs34246903 in 4p16.2 showed a significant effect in both the nose and philtrum region. With the major allele, the nasal ridge is narrowed, the width of the nose tip is decreased and the philtrum is protruded. rs10512248 in 9q22.32 showed an effect on the shape of the nose, where the angle between the nose and the lip decreases toward the major allele. The effect of rs227731 in 17q22 is located in the philtrum, with the minor allele associated with a more protruded philtrum. A short description of knowledge on the significant variants was provided in the Supplementary Text.

## Discussion

This study combined the open-ended phenotyping approach proposed in Claes et al. (2018) with a candidate gene set-up to find possible effects of NSCL/P candidate genes on normal-range craniofacial morphology. To analyze the particular facial region in which the SNP is having the strongest evidence of effect, a global-to-local perspective was introduced by performing a data-driven segmentation (Claes et al., 2018). The segmentation in this study produced more compact segments in comparison to Claes et al. (2018), which is a consequence of the increase in sample size in this study. Another approach for a candidate gene association with facial morphology was described in Claes et al. (2014). Here, candidate variants were also tested on normal facial variation using spatially-dense configurations, but only on a global scale. Using this methodology three out of six SNP associations in this study would not have been discovered since they were only significant in local segments.

The replication strategy in Claes et al. (2018) was adapted toward a meta-analysis design. The meta-analysis was performed in a round-robin fashion, which is an elegant way to combine the power of three different datasets without having to select one as the sole discovery dataset. As expected, the behavior of the meta-analysis resulted in (1) a strengthening of the statistical evidence, if an effect was present in all three datasets, even if only suggestive evidence was found in each dataset separately and (2) a weakening of the statistical noise if a false positive was present in one of the datasets. The inclusion criterion for a significant signal in this study required a SNP to reach the Bonferroni adjusted threshold (8.04 × 10^−6^) in at least one segment for one of the three meta-analyses. To decrease the risk of identifying false positives, the Bonferroni threshold was determined by the number of independent segments, the number of independent genetic variants and, although not completely independent, the three meta-analyses.

In each meta-analysis a different dataset was used as the discovery dataset, this way our results indicate that the effects found were independent of the discovery dataset. Figure 3 shows that our results are influenced by sample size. In particular in the Penn State dataset, which has the lowest sample size (*n* = 1,555), rs7590268, rs34346903, and rs10512248 do not reach significance. Another possible explanation is that the true effects in the different datasets are not the same, for example due to small population differences between the datasets. This idea is analogous to between-study heterogeneity in genetic meta-analyses of quantitative traits (Magosi et al., 2017). In our study, we did not find heterogeneity in the effects found in the British (ALSPAC) and Euro-American (PITT and PENN) populations, this might be because we corrected for stratification within the European population, or because the effects found are in fact the same within the broader European and European-American population. To verify this, the directions of the SNP effects in shape space in each discovery analysis were visually inspected for qualitative coherence. Future analyses should include a quantitative analysis of these directions.

Our study is not the first to investigate the effects of genes involved in the etiology of NSCL/P on normal facial morphology. Two studies each analyzed the same set of 11 SNPs on different sets of phenotypic measurements (Boehringer et al., 2011; Liu et al., 2012). Boehringer et al. (2011) found a suggestive association between rs227731 (17q22) and nose width and the same variant was associated with normal facial morphology in Liu et al. (2012) (the associated phenotypic variables were not reported in this study). In our analysis, this SNP was associated with the shape of the philtrum (Figure 3). Liu et al. also found an association between rs7590268 (2p21) and facial morphology. This variant was associated with the shape of the brow ridge in our study (Figure 4). Both Boehringer et al. (2011) and Liu et al. (2012) found associations between 8q24 and craniofacial morphology. This locus is the strongest NSCL/P GWAS signal in European populations (Birnbaum et al., 2009; Beaty et al., 2010; Murray et al., 2012; Leslie et al., 2015). The association between 8q24 and facial morphology was not observed in this study. As for all variants tested that were not associated, true associations might not have been detected due to low power. Another reason for not finding the 8q24 association might be the difference in the methods applied. In both studies MRI-images and 2D photo images were used, in contrast to the use of 3D-surface images in our study. When correcting for multiple testing in Boehringer et al. (2011), the association between 8q24 and bizygomatic distance was no longer significant, which was discussed in the manuscript. Liu et al. corrected for the 48 phenotypes, but an additional correction for the number of SNPs tested was not considered in determining the threshold for statistical significance. Another difference is that both studies discussed used a limited number of landmarks from which distance measurements were derived. These distances cannot fully capture the complexity of human facial shape. In contrast, our use of spatially-dense quasi-landmarks strongly improves the description of facial morphology (Claes et al., 2012). In the case of Liu et al. (2012), principal components (PCs) were also derived from the sparse landmark configurations. These PCs represent a more complex configuration of facial shape than distances, yet they were examined separately instead of in a multivariate fashion. This preselection of single measurements causes information on the combination of measurements to be lost. In our study, a multivariate framework was applied in which the linear combination of PCs from a facial segment that are in maximal correlation with the SNP being tested are extracted. This way, the PCs can be combined without introducing another multiple testing problem.

Another study analyzing the genetic overlap between NSCL/P genetics and the genetics of normal craniofacial variation was conducted by Howe et al. (2018). Polygenic risk scores (PRS) were calculated from NSCL/P data and subsequently used as the variable of interest in an association with normal facial morphology, in which seven distances based on sparse landmarks were used as phenotypic features. The finding in this study was an association between an increased PRS and a decreased philtrum width. Although the use of PRS is distinct from a candidate gene approach, this study presents additional evidence to the hypothesis that genes involved in the development of NSCL/P have an effect on normal facial morphology as well. More specifically, in both Howe et al. and our study, variations in the philtrum were identified to be associated with NSCL/P susceptibility genes.

Additional evidence for the genetic overlap between cleft genetics and craniofacial morphology genetics lies in the hypothesis of facial endophenotypes for NSCL/P (Weinberg et al., 2008, 2009; Roosenboom et al., 2015). The NSCL/P facial endophenotype shows a retrusion of the midfacial region and a broadening of the upper facial area, which can be an expression of NSCL/P susceptibility genes in unaffected relatives but is also part of normal facial variation. In Weinberg et al. (2009), it was found that unaffected relatives show a decreased philtrum width in comparison to the controls. This result coincides with the association found between an increased cleft PRS and a decreased philtrum width in Howe et al. In our study, the philtrum is more protruded toward the risk allele in rs227731. Although distances are not easy to compare directly with a spatially-dense representation, when looking at the results of rs227731, one can imagine that philtrum protrusion leads to a smaller philtrum width and vice versa.

There is some evidence for an effect of NSCL/P susceptibility genes on the shape of the nose. Boehringer et al. (2011) and Liu et al. (2012) found one NSCL/P associated genetic variant (rs1258763 in 15q13) to be associated with nose width. This result was not replicated in this study. On the other hand, NSCL/P endophenotypic features have been found in the nose by Weinberg et al. (2008, 2009). In our study, most significant effects were found in the central facial area: three in the nose and one in the philtrum. This is not surprising for two reasons. First, when looking at human embryological development, in which the development of the nose and palate are tightly linked. The nose bridge and philtrum are formed by the fusion of the medial-nasal processes, which then fuse with the lateral nasal processes and the maxillary swellings to form the sides of the nose, alae, and the maxillae. The anterior palate is also formed with the fusion of the maxillary and medial-nasal process. The fusion of these prominences requires the coordinated growth of the oronasal prominences in a precise temporal-spatial sequence (Dixon et al., 2011). Thus, the genetic regulation mechanisms involved in lip and palate fusion likely also affects nasal and philtrum morphology. Second, the central facial features show a high genetic determination in genome wide studies (Claes et al., 2018).

The variants in this study were annotated to genes that have been shown to play a role in craniofacial development in previous studies (Table 2, Supplementary Text 1). *PAX7*, of which rs742071 is an intron variant, has been shown to play a role in neural crest development (Basch et al., 2006). Neural crest progenitors give rise to craniofacial cartilage, which interestingly is an important structural component in the tip of the nose, with which this variant was associated (Mansouri et al., 1996; Zalc et al., 2015). Another SNP, rs10512248, is an intron variant of *PTCH1*, which is an important factor in the Hedgehog signaling pathway (Aoto and Trainor, 2015). This pathway plays a fundamental role in craniofacial development in vertebrates (Xavier et al., 2016; Everson et al., 2018). Other variants found in this study were annotated to genes, such as *MSX1* and *NOG*, which have been shown to play a role in craniofacial development (Satokata and Maas, 1994; Anderson et al., 2006). Many of these studies look at gene expression studies during embryonic development in animal models. Although these studies can complement ones like the current study, caution should be taken when comparing animal model studies with our study, since we are investigating different species at different stages (embryological vs. post-natal).

In this study we focused our analyses on participants with European ancestry. To analyze possible population similarities and differences, future analyses should include other populations. Furthermore, only lead SNPs from our literature survey were used, not all loci involved in cleft development have been discovered and this study only included variants discovered in NSCL/P GWAS studies. Thus, the candidate variant list used in this study is incomplete.

For future analyses, it would be interesting to calculate polygenic risk scores (PRS) for NSCL/P, and associate these against normal facial variation in an open-ended phenotyping approach. The PRS capture an individual's genetic propensity toward a trait and thus combines the effect sizes discovered in a GWAS into a univariate score capturing a risk for NSCL/P. Combining the information of multiple markers in one score analogous to this study would result in a facial phenotype associated with NSCL/P risk. Additionally, the risk-score could increase power in comparison to the use of individual SNPs. Another approach would be to calculate a quantitative measurement that indicates to what degree the endophenotype is present in each participant and to use this in a genetic mapping effort, using candidate variants, genome-wide SNPs or PRS. The methodology described in this study allows us to study the effects of genes with a known role in a certain condition on facial morphology. In the future, genes responsible for syndromes with a distinct facial phenotype can also be tested in this framework to see if variations in these genes are responsible for morphological changes in the face.

## Conclusion

Many genetic syndromes and malformations are characterized by a distinctive facial phenotype (Jones et al., 2013). The underlying genes might also be involved in normal craniofacial development (Winter, 1996). Since NSCL/P is one of the most frequent congenital craniofacial malformations, its genetic background could also be involved in normal facial morphology. In this study, the open-ended data-driven phenotyping approach from Claes et al.'s (2018) GWAS on normal-range variation in facial shape was used to study the effects of NSCL/P candidate genes on normal-range craniofacial morphology in a global-to-local perspective. We identified six SNPs involved in NSCL/P with effects on the shape of the nose, chin and philtrum area in a non-clinical population. This study gives insight into the complex genetic architecture of normal-range craniofacial morphology. Furthermore, it provides evidence for the interplay between the genetic background of NSCL/P and craniofacial morphology.

## Data availability statement

The datasets analyzed for this study are available through various sources. For the Pittsburgh dataset, the genotypic markers are available through the dbGaP controlled-access repository (http://www.ncbi.nlm.nih.gov/gap) at accession phs000949.v1.p1. The 3D facial images are available through the FaceBase Consortium (https://www.facebase.org/data/record/#1/isa:dataset/RID=14283) The participants making up the Penn State University dataset were not collected with broad data sharing consent. Given the highly identifiable nature of both facial and genomic information and unresolved issues regarding risk to participants, we opted for a more conservative approach to participant recruitment. Broad data sharing of these collections would thus be in legal and ethical violation of the informed consent obtained from the participants. This restriction is not because of any personal or commercial interests. Additional details can be requested from M.D.S. The ALSPAC data will be made available to bona fide researchers on application to the ASLAPC Executive Committee.

## Ethics statement

Institutional Review Board (IRB) approval was obtained at each recruitment site, and all participants gave their written informed consent before participation; for children, written consent was obtained from a parent or legal guardian. For the Pittsburgh sample, the following local ethics approvals were obtained: University of Pittsburgh IRB PRO09060553 and RB0405013; UT Health Committee for the Protection of Human Subjects HSC-DB-09-0508; Seattle Children's IRB 12107; University of Iowa Human Subjects Office/IRB 200912764 and 200710721. For the Penn State sample, the following local ethics approvals were obtained: State College, PA (IRB 44929 and 4320); New York, NY (IRB 45727); Urbana-Champaign, IL (IRB 13103); Dublin, Ireland; Rome, Italy; Warsaw, Poland; and Porto, Portugal (IRB 32341); Twinsburg, OH (IRB 2503). For the ALSPAC sample, ethical approval for the study was obtained from the ALSPAC Ethics and Law Committee and the Local Research Ethics Committees.

## Author contributions

KI performed all analyses and wrote the first draft of the manuscript under supervision of PC, GH, and HP. PC, JR, EF, JS, SW, MM, JW, and MS conceptualized the design of the study. JR, EF, JS, SW, and MM organized the PITT cohort. JW and MS organized the PSU cohort and imputed the PSU genetic data. SR coordinated the collection of the ALSPAC images. JR, HH, and JW provided input throughout the analyses and the writing process. All authors contributed to manuscript revision, read and approved the submitted version.

### Conflict of interest statement

The authors declare that the research was conducted in the absence of any commercial or financial relationships that could be construed as a potential conflict of interest.

## Footnotes

^1^http://www.bris.ac.uk/alspac/researchers/data-access/data-dictionary/

^2^https://imputation.sanger.ac.uk

^3^Facial mapping software available on https://github.com/TheWebMonks/meshmonk
