# Supplementary material for: Six NSCL/P Loci Show Associations With Normal-Range Craniofacial Variation
Source: Front Genet. 2018 Oct 25;9:502. doi: 10.3389/fgene.2018.00502 (PMC6210408; doi:10.3389/fgene.2018.00502)
Supplement: Supplementary file 1 [file Data_Sheet_1.docx]

Supplementary Material

Four NSCL/P loci associated with normal craniofacial morphology.

Karlijne Indencleef^1,2,*^, Jasmien Roosenboom^2^, Hanne Hoskens^2,4^, Julie D. White^5^, Mark D. Shriver^5^, Stephen Richmond^6^, Hilde Peeters^4^, Mary L. Marazita^3^, Seth M. Weinberg^3^, Greet Hens^7^, Peter Claes^1,2,*^

*** Correspondence:** [karlijne.indencleef@kuleuven.be](mailto:karlijne.indencleef@kuleuven.be), peter.claes@kuleuven.be

# Supplementary Figures


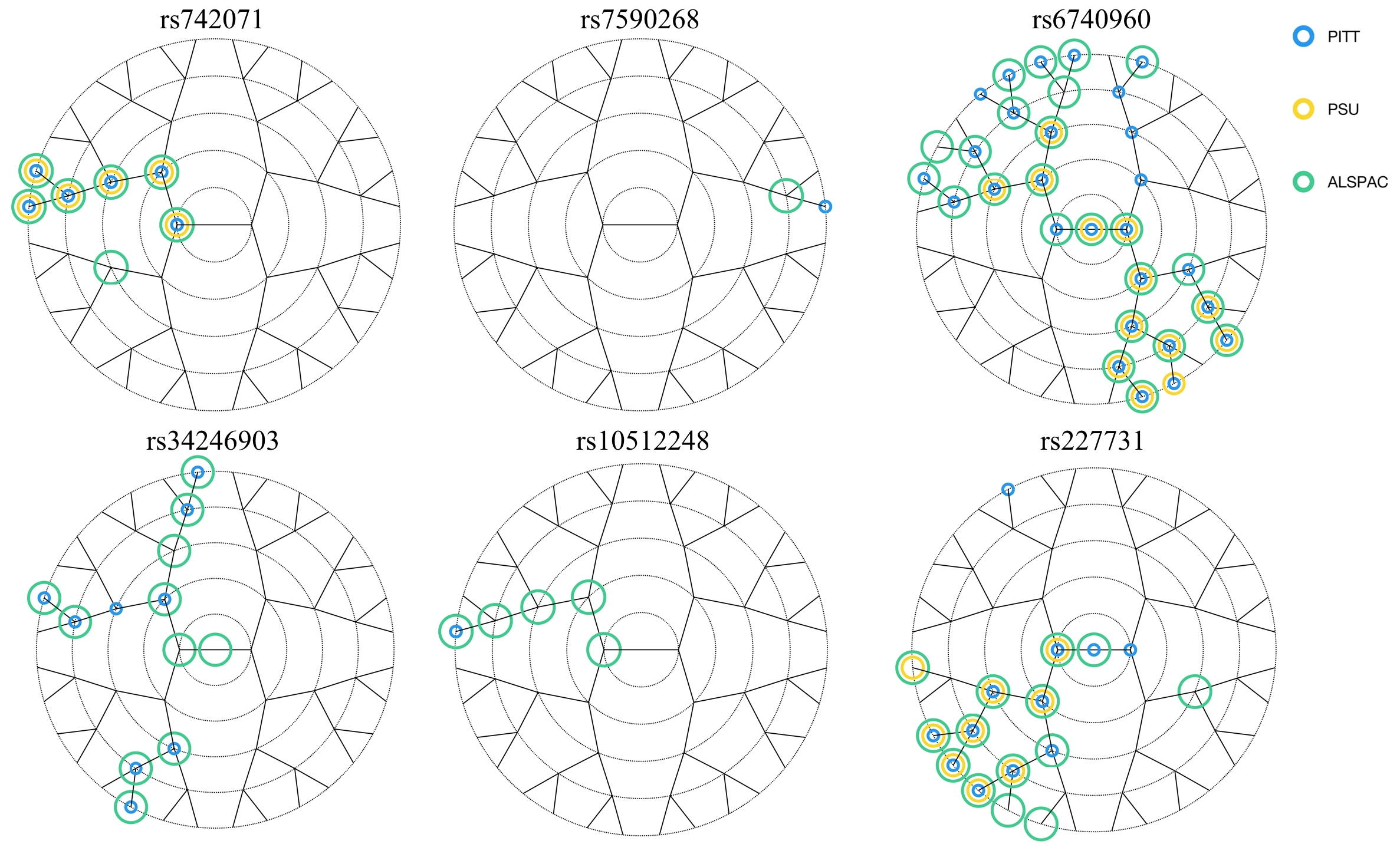


**Supplementary Figure 1: Meta-analysis results. The segments are encircled when significant (p-value < 8.04x10^-6^) in the meta-analysis with the color-coded dataset as discovery dataset.**

1. **Supplementary Text**

## Significant variants in literature

rs742071 in 1p36 is an intron variant of *PAX7*, which encodes a paired box domain transcription factor that plays a role in neural crest development (Basch, Bronner-Fraser, and García-Castro 2006). Neural crest cells are an ectoderm-derived multipotent cell population that gives rise to various cell lineages, including craniofacial cartilage. Mutant mice with impaired Pax7 function display severe craniofacial defects, showing malformations in the facial structures involving the maxilla and nose (Mansouri et al. 1996; Zalc et al. 2015).

rs7590268 is an intron variant of *THADA*, a gene associated with polycystic ovary syndrome and the target of 2p21 chromosomal aberrations in benign thyroid adenomas (Goodarzi et al. 2012; Day et al. 2015; Rippe et al. 2003). In contrast to all other loci mentioned above, this SNP has no previous findings in craniofacial development, except for the association with NSCL/P (Ludwig et al. 2012).

The strongest effect was found for rs6740960 on 2p21, which lies in a non-coding region. This effect was also found by Claes *et al.* (2018), where the SNP was found to be located within an active craniofacial neural crest cell enhancer. The SNP maps 100 kb upstream of *PKDCC*, a possible candidate gene that encodes the cytoplasmic protein kinase domain containing protein. In zebrafish *PKDCC*-orthologs have been shown to be involved in craniofacial development (Melvin et al. 2013). Although both rs7590268 and rs6740960 are localized in 2p21, the effects found in this study were independent. When identifying these SNPs in a NSCL/P case-control GWAS, Ludwig *et al.* (2017) used conditional analysis to show the effects were independent from rs6740960. Additionally, the effects of the two SNPs were located in two different facial areas.

rs34246903 is located 50 kb upstream of *MSX1*, which encodes a transcription factor upregulated during embryogenesis and postnatal development in bone tissue. *BMP* expression in palatal mesenchyme is regulated by the *MSX1* homeobox gene and plays an import role in epithelial-mesenchymal interaction throughout embryogenesis (Satokata and Maas 1994).

rs10512248 is an intron variant of *Patched1* or *PTCH1*. This gene encodes a receptor in the Hedgehog signaling pathway and plays a major role in craniofacial development (Aoto and Trainor 2015).

In 17q22, rs227731 *NOG*, which encodes a BMP antagonist. NOG has been found to play a role in neural crest formation by regulating BMP signaling (Anderson et al. 2006). Matsui & Klingensmith (2014) found evidence in mouse studies that NOG is required in neural crest cells for palatal development, and that *NOG* is expressed during early craniofacial development and acts in neural crest cells to regulate mandible shape and size.

## Supplementary Tables

**Supplementary Table 1: Discovery and meta-analysis results of all SNPs in all segments. Minor allele < major allele. CC, canonical correlation. The CCA-correlations are listed in the order of Pittsburgh, Penn State and ALSPAC, the same order is given for the discovery p-values and the meta-analysis p-values. Supplementary Table in separate Excel File**

**Supplementary Table 2: Linkage Disequilibrium in 1p36 calculated with the NCI NIH analysis tool LDmatrix in LDlink (Machiela and Chanock 2015).**

|  | rs9439713 | | rs4920524 | | rs742071 | |
| --- | --- | --- | --- | --- | --- | --- |
|  | D' | R^2^ | D' | R^2^ | D' | R^2^ |
| rs9439713 | 1.0 | 1.0 | 0.992 | 0.984 | 0.991 | 0.873 |
| rs4920524 | 0.992 | 0.984 | 1.0 | 1.0 | 1.0 | 0.888 |
| rs742071 | 0.991 | 0.873 | 1.0 | 0.888 | 1.0 | 1.0 |

Anderson, Ryan M., Rolf W. Stottmann, Murim Choi, and John Klingensmith. 2006. “Endogenous Bone Morphogenetic Protein Antagonists Regulate Mammalian Neural Crest Generation and Survival.” *Developmental Dynamics* 235 (9): 2507–20. https://doi.org/10.1002/dvdy.20891.

Aoto, Kazushi, and Paul A. Trainor. 2015. “Co-Ordinated Brain and Craniofacial Development Depend upon Patched1/XIAP Regulation of Cell Survival.” *Human Molecular Genetics* 24 (3): 698–713. https://doi.org/10.1093/hmg/ddu489.

Basch, Martín L., Marianne Bronner-Fraser, and Martín I. García-Castro. 2006. “Specification of the Neural Crest Occurs during Gastrulation and Requires Pax7.” *Nature* 441 (7090): 218–22. https://doi.org/10.1038/nature04684.

Day, Felix R., David A. Hinds, Joyce Y. Tung, Lisette Stolk, Unnur Styrkarsdottir, Richa Saxena, Andrew Bjonnes, et al. 2015. “Causal Mechanisms and Balancing Selection Inferred from Genetic Associations with Polycystic Ovary Syndrome.” *Nature Communications* 6 (September): 8464. https://doi.org/10.1038/ncomms9464.

Goodarzi, Mark O., Michelle R. Jones, Xiaohui Li, Angela K. Chua, Obed A. Garcia, Yii-Der I. Chen, Ronald M. Krauss, et al. 2012. “Replication of Association of DENND1A and THADA Variants with Polycystic Ovary Syndrome in European Cohorts.” *Journal of Medical Genetics* 49 (2): 90–95. https://doi.org/10.1136/jmedgenet-2011-100427.

Machiela, Mitchell J., and Stephen J. Chanock. 2015. “LDlink: A Web-Based Application for Exploring Population-Specific Haplotype Structure and Linking Correlated Alleles of Possible Functional Variants.” *Bioinformatics* 31 (21): 3555–57. https://doi.org/10.1093/bioinformatics/btv402.

Mansouri, A., A. Stoykova, M. Torres, and P. Gruss. 1996. “Dysgenesis of Cephalic Neural Crest Derivatives in Pax7−/− Mutant Mice.” *Development* 122 (3): 831–38.

Matsui, Maiko, and John Klingensmith. 2014. “Multiple Tissue-Specific Requirements for the BMP Antagonist Noggin in Development of the Mammalian Craniofacial Skeleton.” *Developmental Biology* 392 (2): 168–81. https://doi.org/10.1016/j.ydbio.2014.06.006.

Melvin, Vida Senkus, Weiguo Feng, Laura Hernandez-Lagunas, Kristin Bruk Artinger, and Trevor Williams. 2013. “A Morpholino-Based Screen to Identify Novel Genes Involved in Craniofacial Morphogenesis.” *Developmental Dynamics : An Official Publication of the American Association of Anatomists* 242 (7): 817–31. https://doi.org/10.1002/dvdy.23969.

Rippe, Volkhard, Norbert Drieschner, Maren Meiboom, Hugo Murua Escobar, Ulrich Bonk, Gazanfer Belge, and Jörn Bullerdiek. 2003. “Identification of a Gene Rearranged by 2p21 Aberrations in Thyroid Adenomas.” *Oncogene* 22 (38): 6111–14. https://doi.org/10.1038/sj.onc.1206867.

Satokata, I., and R. Maas. 1994. “Msx1 Deficient Mice Exhibit Cleft Palate and Abnormalities of Craniofacial and Tooth Development.” *Nature Genetics* 6 (4): 348–56. https://doi.org/10.1038/ng0494-348.

Zalc, Antoine, Revital Rattenbach, Frédéric Auradé, Bruno Cadot, and Frédéric Relaix. 2015. “Pax3 and Pax7 Play Essential Safeguard Functions against Environmental Stress-Induced Birth Defects.” *Developmental Cell* 33 (1): 56–66. https://doi.org/10.1016/j.devcel.2015.02.006.
